# Specificity of H_2_O_2_ signaling in leaf senescence: is the ratio of H_2_O_2_ contents in different cellular compartments sensed in *Arabidopsis* plants?

**DOI:** 10.1186/s11658-021-00300-w

**Published:** 2022-01-06

**Authors:** Ulrike Zentgraf, Ana Gabriela Andrade-Galan, Stefan Bieker

**Affiliations:** ZMBP (Centre of Plant Molecular Biology), University of Tübingen, Auf der Morgenstelle 32, 72076 Tübingen, Germany

**Keywords:** Leaf senescence, Free oxygen radicals, ROS, Hydrogen peroxide, Stromules, Senescence regulation, Intracellular compartments

## Abstract

Leaf senescence is an integral part of plant development and is driven by endogenous cues such as leaf or plant age. Developmental senescence aims to maximize the usage of carbon, nitrogen and mineral resources for growth and/or for the sake of the next generation. This requires efficient reallocation of the resources out of the senescing tissue into developing parts of the plant such as new leaves, fruits and seeds. However, premature senescence can be induced by severe and long-lasting biotic or abiotic stress conditions. It serves as an exit strategy to guarantee offspring in an unfavorable environment but is often combined with a trade-off in seed number and quality. In order to coordinate the very complex process of developmental senescence with environmental signals, highly organized networks and regulatory cues have to be in place. Reactive oxygen species, especially hydrogen peroxide (H_2_O_2_), are involved in senescence as well as in stress signaling. Here, we want to summarize the role of H_2_O_2_ as a signaling molecule in leaf senescence and shed more light on how specificity in signaling might be achieved. Altered hydrogen peroxide contents in specific compartments revealed a differential impact of H_2_O_2_ produced in different compartments. *Arabidopsis* lines with lower H_2_O_2_ levels in chloroplasts and cytoplasm point to the possibility that not the actual contents but the ratio between the two different compartments is sensed by the plant cells.

## Introduction

Senescence is a phase of plant development and becomes visible by the degreening of leaves, in which the photosynthetic apparatus is dismantled and chlorophyll is broken down, leading to light green and yellowish leaves [1]. However, when the light green color becomes apparent, the senescence program has been initiated long before, and changes at the molecular and physiological level have already been executed [2]. Plant cells must integrate a myriad of different signals driving onset and progression of senescence [3]. In the extreme scenario the already started program can be stopped or even reversed [1]. Even though senescence is often designated as the last step in the plant’s life history, it may occur from relatively early on throughout development. Older leaves in the lower canopy which are shaded by newly formed leaves or neighboring plants, or simply have reached a certain age, are sacrificed for the sake of newly developing organs, for example new leaves or stems. The aim of the senescence program is the remobilization of previously acquired resources such as nitrogen, carbon and mineral compounds out of the senescing tissues into developing parts of the plant. After remobilization has been executed, the senescent organs finally die and are shed. Before anthesis, this process is called sequential leaf senescence and aims at the repartitioning of nutrients from older leaves to newly developing, non-reproductive organs. In this case, the senescence program is restricted to a single organ. After anthesis, monocarpic senescence sets in and nutrients are now reallocated to the newly developing reproductive organs such as siliques and seeds. After this transition to reproductive growth during monocarpic senescence, potentially all leaves of a plant can now execute senescence, which finally leads to the death of the whole plant except for the newly formed seeds [4]. This systemic onset has to be coordinated between the different organs. However, how the local and systemic leaf senescence programs are discriminated is largely unknown. Even though a master regulator for leaf senescence was intensively searched for, it became clear that such a regulator does not exist. In contrast, multi-layered complex regulatory networks are in place to drive onset, progression and, in extreme cases, reversal of leaf senescence [3, 5]. For example, the transcription factor WRKY53 is just one of more than a hundred transcription factors driving the onset of senescence. Solely this transcription factor is tightly regulated at several levels. Not only is its expression responsive to ROS, jasmonic or salicylic acid (JA and SA) and influenced by more than 12 other transcription factors, also its activity is regulated by different molecular mechanisms, e.g. phosphorylation by MAPKKK1 or by changing interaction partners. On top of that, the degradation of the WRKY53 protein is controlled though a HECT ubiquitin ligase (UPL5) which exhibits an opposite expression pattern compared to WRKY53 itself. This ensures that even if the gene is mis-expressed for whatever reason, the protein content is still controlled by degradation and the protein will only be stable in the cells during onset of senescence [5]. To make the story even more complicated, WKRY53 also has a role in pathogen and wounding response. The overlap between senescence and pathogen response has been recognized in many instances due to similar signals which appear to be used in both processes. Recently, the cross-regulation network between leaf senescence and plant immunity, which is mediated by SA and ROS, has been nicely summarized [6].

Moreover, the senescence program adapts to diverse environmental conditions and requires very high plasticity. All kinds of stresses, abiotic as well as biotic, are sensed and constantly integrated into the regulatory networks, in which long-lasting unfavorable conditions have the potential to induce senescence prematurely [6, 7]. This serves as an exit strategy to guarantee viable offspring even under stressful conditions. As a tradeoff, seed quantity and quality are often diminished and can significantly lower productivity of plants. Consequently, in crop plants, stress-induced premature senescence can have a major negative impact on yield quantity and quality. Even though our knowledge on the physiological changes has increased enormously during the last decades and more and more signaling molecules acting in this process have been characterized, we are still far from understanding how this complex program is coordinated and how specificity is achieved in signaling. Almost all plant hormones are involved in regulation of this process as well as smaller signaling molecules such as calcium or ROS. All these components also have roles in other processes, so the question how specificity is achieved, especially in the case of ROS signaling, is not yet answered. How does the cell differentiate between an oxidative burst elicited by pathogen infection and the oxidative burst coinciding with the induction of senescence? In this review letter, we would like to shed light on the role of the ROS hydrogen peroxide in senescence signaling with a focus on the differences in signaling of H_2_O_2_ produced in different organelles. Even though hydrogen peroxide appears to be used as a signaling molecule in many different plant species, we focus on the findings in the model plant *Arabidopsis thaliana*.

## General alterations in different organelles during senescence

In contrast to other cell death reactions, the long lasting and complex regulatory process to adequately execute leaf senescence requires continuous control by the nucleus as well as a constant energy supply. Consequently, not only is the nucleus kept intact until late stages of senescence, but also the mitochondria need to stay active to deliver the energy driving the process. Functionality of both organelles has to be guaranteed during onset and progression of senescence until finally both are also degraded in the terminal phase. In contrast, chloroplasts are massively restructured during progression of senescence and lose their photosynthetic function while peroxisomes are converted back to glyoxysomes with changed functional properties.

### Chloroplasts

Chloroplasts are converted into “gerontoplasts” during the progression of leaf senescence. These gerontoplasts are morphologically characterized by an increased number of enlarged plastoglobuli, disorientation of the grana stacks and swelling of the thylakoids [1, 8]. These structural changes are combined with loss of function of the photosynthetic apparatus, thus allowing chlorophyll loss and photochemical capacity (F_V_/F_M_) to be used as parameters to describe senescence. As the majority of assimilated nutrients are stored in chloroplasts in the form of photosynthetic proteins, the remobilization of chloroplastic components is a central feature of senescence. It was estimated in the 1980s that more than 10 billion tonnes of Rubisco and 1 billion tonnes of chlorophyll are produced and degraded every year [9]. Even though removal of damaged proteins from chloroplasts takes place throughout plant development as part of the quality control of chloroplasts, chloroplast dismantling and degradation of chloroplastic proteins is strongly upregulated during senescence. Besides various types of chloroplastic proteases acting inside the chloroplasts, diverse extra-plastidic pathways mediate degradation of chloroplastic proteins. Intensive formation of different vesicles can be observed during senescence ranging from RCBs (Rubisco-containing bodies) and ATG8-interacting protein1-plasmid associated (ATI-PS) bodies, which depend on the autophagy machinery for further degradation, to SAVs (senescence-associated vacuoles) and CCVs (chloroplast vesiculation-containing vesicles) acting independently of autophagy [10, 11]. Moreover, whole chloroplasts can be degraded by “chlorophagy” [12] or by the 26S proteasome mediated by a cytosol-localized E3 ubiquitin ligase mainly targeting chloroplasts that over-accumulate singlet oxygen [13]. Degradation processes inside and outside the chloroplasts have to be coordinated and different pathways are induced at distinct time points during leaf senescence, e.g. chloroplast number is reduced at the later stages [11].

### Peroxisomes

Peroxisomes consist of an amorphous matrix surrounded by a single membrane. Notably, the term “peroxisomes” originates from their high H_2_O_2_ content. A significant number of enzymatic systems capable of generating H_2_O_2_ are present in the peroxisomes, e.g. acyl CoA oxidase, glycolate oxidase, superoxide dismutase, urate oxidase and many others. The extensive production of H_2_O_2_ is counterbalance by the presence of large amounts of the H_2_O_2_ scavenging enzyme catalase. Besides being generated as a side product of various reactions, H_2_O_2_ itself has a critical role as a signal molecule in the crosstalk between peroxisomes and other organelles to coordinate cellular functions. Apart from the peroxisomal metabolism of ROS, reactive nitrogen species and reactive sulfur species with signaling functions are produced in peroxisomes [14]. Moreover, peroxisomes also have important roles in the biosynthesis of plant hormones, such as JA, indole-3-acetic acid (IAA) and SA, all of which influence leaf senescence in one way or another. Therefore, peroxisomes are important signal generators in the cell. Not only do peroxisomes undergo dynamic changes in metabolism and morphology, but also peroxisome abundance can be adjusted to environmental conditions. Moreover, the primary functions of plant peroxisomes vary with different developmental stages. Whereas peroxisomes in green leaves are mainly involved in photorespiration, peroxisomes in seeds harbor the glyoxylate cycle together with β-oxidation and are therefore called glyoxysomes. In senescent tissues, peroxisomes are converted into “gerontosomes” which resemble glyoxysomes in terms of their metabolic functions [14, 15].

### Mitochondria

Even though mitochondria exhibit a driving force in programmed cell death (PCD) in animal systems, in which the loss of the mitochondrial membrane integrity leads to the release of PCD elicitors such as cytochrome C, plant mitochondrial membranes are maintained until very late stages of senescence. Thus, it is unlikely that plant mitochondria will trigger plant senescence in a similar way as they do during animal PCD. In *Arabidopsis*, functionality of the mitochondria is preserved until the latest stages of leaf senescence, even though their number drops by 30%. In contrast to structural changes observed in chloroplasts, the ultrastructure of the remaining mitochondria appears to be unchanged, retaining clearly demarcated cristae and membrane boundaries throughout the process of senescence. Integrity of mitochondria as well as their ability to provide energy are maintained during leaf senescence to drive the energetically demanding reallocation of nutrients. Transcriptome analysis combined with metabolomic approaches revealed that mitochondrial metabolism is partially reorganized to support the selective catabolism of both amino acids and fatty acids. Thereby, mitochondria provide the carbon backbone essential for nitrogen remobilization, which is of utmost importance during senescence [16]. In parallel, the alternative respiration pathway is activated during senescence [17]. An important function of the alternative oxidase (AOX) is to prevent the formation of excess ROS. Hence, a low reduction status of the ubiquinone pool by oxidizing ubiquinol is ensured, and thus the electron flow is guaranteed [18].

### The nucleus

The nucleus appears to be the command center for senescence coordination. Therefore, its overall structure and integrity are maintained to guarantee its function. However, onset and progression of this developmental process is driven by massive changes in gene expression; e.g. in *Arabidopsis thaliana* almost one quarter of all genes are differentially regulated during senescence [2]. While genes involved in degradation and mobilization of macromolecules are switched on, genes related to photosynthesis are turned off. A chronology of events has been determined by high-resolution temporal transcript profiling covering 22 time points of single leaves of a defined position within *Arabidopsis* rosettes [2]. In this chronology, activation of autophagy and transport as well as the response to ROS are prominent early events of leaf senescence. These massive changes in the transcriptome imply a central and important role for transcriptional regulators [19, 20]. Two transcription factor (TF) families, the WRKY and the NAC factors, which greatly expanded in the plant kingdom, appear to play outstanding roles in senescence regulation in *Arabidopsis* but also in other plant species [21, 22]. NAC proteins form one of the largest plant-specific TF super families, and its members control transcriptional reprogramming associated with many developmental processes including senescence [22, 23]. Recently, genome-wide dynamic modelling of transcriptional gene regulatory networks uncovered new and distinct pathways during the onset of *Arabidopsis* leaf senescence including miRNA action [24]. Besides the expansion in number, the WRKY family adopted a specific functional strategy of networking. Almost all *Arabidopsis* WRKY transcription factor genes are characterized by one or several binding motifs for WRKY factors, so called W-boxes, in their promoters, implying a complex WRKY-driven transcriptional network [25]. Moreover, complexity is increased even more by the fact that WRKY factors are also able to form homo- and heterodimers with different functional properties [26–28].

## A long-lasting H_2_O_2_ increase acts as a signal in leaf senescence of ***Arabidopsis***

In the absence of stress, developmental senescence is mainly driven by the actual age of the individual leaves and the age and developmental stage of the whole plant [29]; however, the mechanisms by which plants sense these parameters is still not well understood. The senescence program is synergistically as well as antagonistically influenced by almost all plant hormones [3, 20]. Moreover, during the last two decades, it became evident that ROS, and especially hydrogen peroxide, can function as signaling molecules in developmental as well as stress-induced senescence. On the one hand, ROS can have a direct impact on gene expression by altering the expression as well as the activity of transcription factors [2, 30–33], thereby modulating regulatory processes. On the other hand, ROS can oxidize more or less all kinds of macromolecules, influencing a variety of physiological changes. Less well-known signaling compounds such as melatonin are involved in postharvest senescence regulation by modulating the ROS homeostasis [34].

ROS are inevitable by-products of aerobic metabolism in all organisms. They are formed by energy transfer to or partial reduction of molecular oxygen (O_2_) producing either free radicals such as superoxide (O^**·**−^_2_) and hydroxyl radicals (OH^**·**^) or non-radical but highly reactive molecules such as hydrogen peroxide (H_2_O_2_) and singlet oxygen (^1^O_2_), of which the latter is generated exclusively in the chloroplasts. Here, excess light energy results in an electron spin shift in the molecular orbitals, which renders ^1^O_2_ very unstable and highly reactive. Except H_2_O_2_, which has a half-life of milliseconds to seconds [35] and can traverse longer distances, including across plant cell membranes via aquaporins [36], all ROS have very short half-lives and relatively high oxidation potentials, and thus act close to their site of production. All ROS can act as signaling molecules but, when present in excess, are toxic for the cell. Therefore, a delicate balance between ROS production and scavenging needs to be established in all cellular compartments. OH^**·**^ is the most reactive and thus most toxic ROS. Despite the apparent lack of an established system for the tight control of hydroxyl radicals, its function in multiple processes has been documented as e.g. ion flux in roots, germination, stomatal closure, or post-harvest senescence of fruits [37–40]. Most recently, Hancock and Russel [41] suggested a H_2_-dependent scavenging mechanism for OH^**·**^. However, the reactive and short-lived nature of OH^**·**^ renders its study challenging. In any case, the cell should avoid the Fenton reaction between H_2_O_2_ and O^**·**−^_2_ which produces OH^**·**^ in the presence of metal, especially iron, ions [42]. The inherent reactivity of different ROS determines their reactivity towards specific sites in all kinds of proteins. Whereas due to its high oxidative potential OH^**·**^·does not pick and choose, the superoxide anion (O^**·**−^_2_) predominately attacks iron-sulfur ([Fe-S]) clusters while H_2_O_2_ targets Cys residues. Therefore, the subcellular colocalization of short-lived ROS and their respective targets might contribute to the ROS signaling specificity [43].

**Fig. 1:**
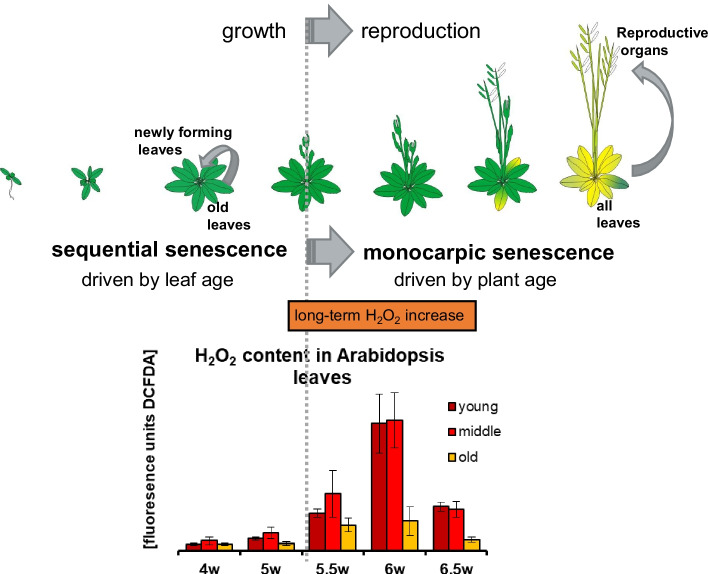
A schematic drawing of the development of the model plant *Arabidopsis thaliana* is shown at the top. At the transition from growth to reproduction (dotted grey line), senescence switches from sequential senescence which remobilizes nutrients from old to young leaves to monocarpic senescence in plants which started to bolt and flower. Now nutrients are remobilized from all rosette leaves to the now developing reproductive organs. During the time interval of bolting and flowering, intracellular hydrogen peroxide contents were measured and are indicated in arbitrary units of DCFDA fluorescence. H_2_O_2_ levels increased during transition to reproductive growth and the long-term increase at the transition point is indicated by the orange bar. H_2_O_2_ concentration showed more pronounced elevations in young (dark red; pos. 8–12 within the rosette) and middle-aged leaves (red, pos.4–7 within the rosette) than in old leaves (orange, pos. 1–3 within the rosette). The age of the plants is indicated in weeks after sowing (w)

Similar to plant hormones, ROS participate in a wide range of developmental and stress-related signaling processes, leaving the question largely open, how specificity can be achieved, how the plant cells can differentiate signaling cues and elicit an appropriate response. So far, no clear signature, e.g. the amplitude, frequency or duration of Ca^2+^ spikes [44], can be linked to ROS or more specifically to H_2_O_2_ signaling. In contrast to calcium signaling, which is executed by storage and release of Ca^2+^ [45], ROS signaling is controlled by production and scavenging. In *Arabidopsis*, a network of at least 152 genes is involved in managing the ROS levels [46]. However, in contrast to e.g. the oxidative burst after pathogen infection or wounding lasting for minutes to hours [42], we observed a clear difference in the duration of the senescence-related H_2_O_2_ signal, lasting for more than a week during induction of monocarpic senescence, at least in *Arabidopsis* or oil-seed rape [47, 48]. Measurement of intracellular H_2_O_2_ contents in different leaves of a single *Arabidopsis* rosette indicated that the increase during onset of monocarpic senescence can be observed more prominently in the younger leaves (Fig. 1) [48], suggesting that sequential senescence might use different signals.

This long-term intracellular H_2_O_2_ increase at the onset of monocarpic senescence in *Arabidopsis* and oilseed rape is predominately due to sophisticated regulation of the activities of the H_2_O_2_ scavenging enzymes catalase (CAT) and ascorbate peroxidase (APX) [48, 49]. All subcellular compartments are equipped with their own enzymatic and non-enzymatic scavenging systems. While catalases are predominantly located in the peroxisomes, different APX isoforms are found in chloroplasts, mitochondria, the cytosol and the outer membrane of the peroxisomes. Catalases have a very low affinity for H_2_O_2_, with a K_m_ of approx. 43 mM, but have a high reaction rate and are highly abundant, which compensates for this low affinity [50]. Under oxidative stress conditions, CAT can be retained in the cytosol to protect this compartment against H_2_O_2_-mediated redox changes and intensify defenses against oxidative damage [51]. In *Arabidopsis*, APX3 and 5 isoforms are associated with the outer peroxisomal membrane. This localization and the much higher affinity of APXs to H_2_O_2_ as compared to CAT (100 μM), could be responsible for the tight control of H_2_O_2_ leakage from peroxisomes to the cytosol [52–55]. Furthermore, APX1, 2 and 6 are present in the cytoplasm and two additional APX isoforms are associated with the stroma (sAPX) and the thylakoid membranes (tAPX) of the chloroplasts. Therefore, CAT and APX are positioned strategically to tightly control H_2_O_2_ concentrations and to ensure that H_2_O_2_ can act as a signaling molecule [56].

To allow for a long-term intracellular H_2_O_2_ increase at the onset of monocarpic senescence, in the first place *CAT2* gene expression starts to decline at bolting time through inhibition of transcription by the bZIP transcription factor G-Box binding factor 1 (GBF1). This factor is most likely activated by phosphorylation through casein kinase II and translocation to the nucleus [48, 57–59]. As CAT2 protein has a high turn-over rate and accounts for approximately 80% of the total CAT activity in leaves [60], this rapidly leads to a decline in CAT2 enzyme activity and an increase in H_2_O_2_ concentration. In addition, APX1 activity also declines at the same time during bolting and flowering, but its activity is restored at later time points. In this case, gene expression is not downregulated but inhibition of activity appears to be carried out post-transcriptionally [61]. All in all, this leads to low protection against H_2_O_2_ in the cytosol as well as in the peroxisomes. Miyake and Asada [62] found that under low concentrations of ascorbate, hydrogen peroxide damages compound I of ascorbate peroxidase and inactivates enzyme activity. Furthermore, an experiment with suspension culture cells also showed that, paradoxically, APX activity can be inhibited by its own substrate H_2_O_2_. H_2_O_2_ treatment of whole plants revealed that this inhibition can only be induced during the time window of bolting and flowering, and exogenous application of H_2_O_2_ is only effective during this period [48]. Moreover, removal of the stem leads to restoration of APX activity, and subsequent re-bolting induces a second period of APX inactivation [63], clearly indicating a connection of the inhibitory mechanism to the bolting and flowering state. This means that APX is rendered sensitive towards hydrogen peroxide by a so far unknown mechanism during this specific period. Thus, at this time point, H_2_O_2_ concentrations further increase and create a positive feedback loop. After a certain time interval, inhibition of APX is released again and, in addition, *CAT3* expression and enzyme activity start to increase. This regulatory cue results in a long-term hydrogen peroxide elevation as illustrated in Fig. 1. If downregulation of *CAT2* expression and activity is abolished in *gbf1* knock-out plants, APX activity is not inhibited by increasing H_2_O_2_ amounts, no long-term H_2_O_2_ increase is created and the onset of senescence is delayed [57]. This clearly indicates that this long-lasting H_2_O_2_ elevation acts as a signal to induce monocarpic senescence.

Furthermore, so far only scant attention has been dedicated to the fact that the circadian clock also triggers transcriptional regulation of ROS-responsive genes, ROS homeostasis, tolerance to oxidative stress [64–66] as well as senescence [67]. Not only do hydrogen peroxide production and scavenging display time-of-day phases but also mutations in the core-clock regulator, CIRCADIAN CLOCK ASSOCIATED 1, as well as mis-expression of components of the evening complex, EARLY FLOWERING 3, LUX ARRHYTHMO, and TIMING OF CAB EXPRESSION, affect ROS production [67]. This clearly indicates a global effect of the clock on the ROS network, and hence Sanchez and Kay [68] have suggested calling the circadian clock the “mastermind” of plant life. Interestingly, the period of the diurnal rhythm also changes during senescence, decreasing from 24 h in young plants to 22–23 h in old plants [69]. Therefore, time of the day should always be considered in future research.

## Impact of H_2_O_2_ generated in different subcellular compartments in ***Arabidopsis*** leaves

In the last two decades it has become clear that the ROS concentrations in the cytoplasm and in different organelles have a different impact on senescence. Changing the H_2_O_2_ levels in different subcellular compartments has different effects on the senescence program ([49, 70], unpublished observations). Increasing H_2_O_2_ production in peroxisomes or in chloroplasts induced two different types of responses, one of which is independent and the other is dependent on the site of H_2_O_2_ production. While the independent response comprised the activation of more general oxidative stress response genes, an H_2_O_2_ increase in chloroplasts specifically induced early senescence signaling components, including transcription factors and biosynthetic genes involved in production of secondary signaling molecules [70]. In 2013, Rosenwasser et al. established a bioinformatic tool called “ROSMETER” for the identification of transcriptomic signatures related to ROS type and production site [71]. Interestingly, an unexpected but highly significant ROS transcriptome signature of mitochondrial stress was detected during the early stages of leaf senescence by the ROSMETER when the high-resolution transcriptome dataset of leaf senescence in *Arabidopsis* of Breeze et al. [2] was analyzed. This signature was defined by different reference plants: (i) rotenone treated plants and (ii) *AOX1*-T-DNA insertion lines under mild drought and light stress and (iii) antisense *AOX1a* plants. Rotenone treatment inhibits complex I of the respiratory electron transport chain and leads to induction of the AOX pathway and thereby to the reduction of ROS production [72, 73], while *AOX1*-T-DNA insertion as well as antisense *AOX1a* expression abolishes the alternative respiration pathway and would therefore increase mitochondrial ROS production, at least under stress conditions. However, there was a strong positive correlation of the early senescence expression profiles with rotenone treatment starting at 23 days after sowing and almost at the same time, from day 25 on, a negative correlation with *AOX1*-T-DNA insertion. This would mean that the transcriptome signature at early stages of leaf senescence would point to low mitochondrial ROS production. However, long-term antimycin A treatment which inhibits complex III of the respiratory chain and induces the AOX pathway or overexpression of *AOX1a* diminished ROS production but did not significantly alter senescence [74]. Therefore, the role of mitochondrial ROS in leaf senescence is still debatable. However, antimycin A also has effects on the cyclic electron flow in the chloroplasts [75].

Peroxisomes appear to be the most oxidized cellular organelles, with a redox potential of approximately − 360 mV [76]. Peroxisomes are one of the main sources of intracellular ROS generation. In photosynthetic tissue, photorespiration is the main source of H_2_O_2_ production in peroxisomes, contributing up to 70% to the total H_2_O_2_ production in plant cells [42]. In addition, photorespiration needs coordination of the chloroplasts, peroxisomes, and mitochondria in the cytosol, and often close contacts of these organelles can be observed, most likely to directly exchange substrates. Interestingly, at these contact sites H_2_O_2_ accumulation was observed inside the peroxisomes, also pointing to a role of H_2_O_2_ in organelle communication [77]. Using in vivo imaging with fluorescent proteins targeted to peroxisomes, rapid formation of tubular extensions called peroxules was observed in response to changes in ROS levels. Not only could these peroxules participate in the transfer of metabolites to mitochondria and chloroplasts, they could also be involved in ROS flux between these compartments, even though so far there is no direct evidence for this assumption [56]. In contrast, similar dynamic structures in chloroplasts called stromules can transfer H_2_O_2_ from chloroplasts to nuclei [78, 79]; however, to date no contact of peroxules to nuclei has been described. To counterbalance the large amounts of H_2_O_2_ generated in peroxisomes, 10–25% of the total peroxisomal proteins are catalases [80], in which CAT2 accounts for approximately 80% of the total catalase activity. As already mentioned above, *CAT2* regulation is a central element of creating the long-lasting increase of intracellular H_2_O_2_ at bolting and flowering time, which is used as a senescence signal [48, 57]. Altering peroxisomal H_2_O_2_ by different means induces changes in gene expression [65, 66, 81, 82] that differ from those induced by chloroplast-derived H_2_O_2_ [70]. Thus, peroxisomal H_2_O_2_ clearly participates in retrograde signaling to the nucleus. However, the underlying molecular mechanisms and crosstalk with ROS from other compartments still have to be elucidated [56, 83]. How this specificity is produced downstream of the production is unclear. Thiol-based antioxidants such as peroxiredoxins, thioredoxins, and glutaredoxins are likely to be involved in ROS signaling cascades [84]. However, H_2_O_2_ can leak out of all compartments to the cytosol, so the cytosol most likely acts as a key site of redox signal integration. Thus, MAPK pathways in the cytoplasm are proposed to integrate ROS signals from different organelles to regulate the gene expression in the nucleus [85].

Like peroxisomes, plastids form stroma-filled tubular extensions surrounded by the envelope membrane called stromules. Stromules are dynamic structures that extend along actin microfilaments and the ER. In chloroplasts, stromule frequency increases during the day or in response to light-sensitive redox signals [86]. Interestingly, stromules can still form on isolated chloroplasts after extraction from the cytoplasm, pointing to the fact that stromule formation is plastid autonomous. In addition, plastid-derived vesicles are suggested to bud from stromules through tip shedding or simple breakage, either to recycle plastid content during nutrient stress, to remove toxic molecules, or for intracellular communication [87]. These stromules allow the plastid compartment to make direct contacts to other subcellular compartments such as other plastids, mitochondria or nuclei. Metabolites as well as proteins can flow through these connective tunnels. Moreover, using a nuclear-targeted H_2_O_2_ sensor protein (HyPER) [88], the translocation of ROS generated in chloroplasts into adjacent nuclei could be observed [89]. This suggests that stromules have a function in retrograde signaling in channeling and speeding up signal transduction directly to the nucleus, avoiding the scavenging systems of the cytoplasm [89]. Additionally, organellar H_2_O_2_ can oxidize thiol groups of specific proteins, thereby converting the ROS signal into thiol redox signals [90, 91].

In addition, changes in the apoplastic space appear to be involved in signaling and molecular trafficking during senescence [92]. Along with variations in the volume and pH of the apoplastic fluid, leaf senescence-related changes in the secretome included many proteins involved in stress responses and ROS metabolism, indicating that leaves of different ages control stress responses differentially to balance growth or survival at the whole plant level [93]. Moreover, production of apoplastic ROS can influence ROS generation in the chloroplast and both together can have an impact on gene expression in the nucleus [94].

On top of that, all these forms of retrograde signaling between the different organelles and the nucleus have to be integrated to finetune the final outcome of gene expression. We are far from understanding which molecular mechanisms underly this crosstalk. Recently, the protein RADICAL-INDUCED CELL DEATH1 (RCD1) has been characterized as a molecular component that allows a dialog between the retrograde signals of both mitochondria and chloroplasts. On one hand, RCD1 directly interacts and inhibits ANAC013 and ANAC017, two regulatory proteins that usually activate a set of genes involved in repair of mitochondria. On the other hand, the nuclear-localized RCD1 changes abundance, redox state and oligomerization in response to ROS generated in the chloroplasts. Interestingly, the interaction domain with the NAC factors was also necessary for the sensitivity to chloroplastic ROS [95].

The search for ROS receptors is far from complete and still has to be continued. It can be speculated that a plethora of ROS sensors is likely to exist in numerous compartments including the apoplast, cytosol, and organelles. In addition, the respective down-stream signaling components are yet to be identified. Plant cells may be able to decode specific ROS-dependent signatures by monitoring the relative intensity of redox stimuli at different locations. Such signatures could be part of the activation of appropriate responses [85].

## In vivo imaging using fluorescent dyes and sensor proteins

Colorimetric methods using nitro blue tetrazolium (NBT) and/or diaminobenzidine (DAB) were initially used to detect ROS in cells, tissues, and organs. However, these methods were not suitable to analyze dynamic ROS levels in vivo. To solve this problem, a collection of different fluorescent dyes was established, in which different dyes exhibit different properties with regard to specificity and cell permeability, allowing in vivo imaging in real time. From among different fluorescent probes which were tested for in vivo imaging (DHE, H2DCFDA, H2HFF-OxyBURST, Amplex red, SOSG, and PO1), H2DCFDA displayed the highest signal-to-noise ratio [96]. One of the drawbacks of these dyes is that often the reactions between ROS and dye are not reversible and the dyes have to be reapplied. In parallel, transgenic approaches were developed using ROS-responsive promoters coupled to reporter genes [97, 98], which also allow one to measure ROS contents at the whole plant level and to follow up systemic ROS signaling. Moreover, protein-based fluorescent sensors have been developed and by now a variety of genetically encoded fluorescent redox probes are available [99]. For example, the redox-sensitive roGFP [100] or the H_2_O_2_ sensitive HyPER [88] can image more precisely the in vivo redox conditions and/or ROS concentrations in cells utilizing ratiometric measurements with two different wavelengths of the excitation laser light. These protein-based approaches also opened up the possibility to direct the proteinaceous sensors to different subcellular compartments via signal peptides or other localization signals. In vivo imaging using these sensor proteins can now be employed to resolve the spatiotemporal dynamics inside the cells and even in different subcellular compartments. Very recently the application of this technique could demonstrate that ROS locally generated in chloroplasts of intact *Arabidopsis* seedlings by methyl viologen treatment cause changes in H_2_O_2_ and glutathione redox potential in other subcellular compartments such as the cytosol and mitochondria [101]. However, given the nature of these sensors, one has to keep in mind that the expression of such sensor proteins cannot only be used to measure in vivo ROS concentrations but at the same time scavenge ROS and thereby also influence intracellular ROS concentrations. When using these sensor lines for long-term studies of ROS levels during senescence, we observed differences in production in different compartments but encountered the problem that all these lines showed altered senescence phenotypes compared to non-transgenic lines ([49], unpublished observations). Therefore, the use of these sensor proteins for in vivo imaging during senescence has its limitations.

However, these lines were still useful to characterize the role of ROS, especially H_2_O_2_ during senescence. For sensing especially H_2_O_2_ the fluorescent protein HyPER was constructed_,_ which combines the regulatory domain of the *Escherichia coli* OxyR transcription factor for sensing H_2_O_2_ in bacteria with a circular permutated YFP [88]. The *E. coli* OxyR transcription factor contains an H_2_O_2_-sensitive regulatory domain (amino acids 80–310, OxyR-RD), and a DNA-binding domain (amino acids 1–79). The reduced form of OxyR-RD is oxidized preferentially by H_2_O_2_. [102]. Aslund et al. suggested that OxyR acts as a peroxidase as OxyR returned to its reduced form in a time course experiment and a significant amount of NADPH was consumed in in vitro experiments [103]. Moreover, canonical H_2_O_2_ measurements using H2DCFDA [49] as well as H_2_O_2_ treatments of HyPER expressing leaf material in a perfusion chamber directly under the confocal LSM revealed that these sensor proteins scavenge H_2_O_2_ (unpublished observations). This means that even though the reaction is reversible, so these proteins can function as sensors, HyPER expression in plants will change the amplitude of intracellular H_2_O_2_ signals by consuming H_2_O_2_. This is exactly what has been observed during onset of monocarpic senescence; the H_2_O_2_ signal in the HyPER expressing lines was dampened and senescence was affected [49]. Having shown this, we now used these lines no longer as sensor lines but as lines with altered intracellular ROS levels as described in the next paragraph.

## Lowering H_2_O_2_ contents specifically in different subcellular compartments

**Fig. 2:**
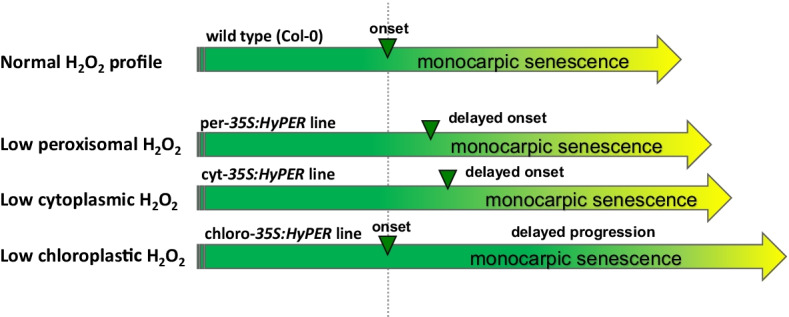
Changes of intracellular H_2_O_2_ contents in different compartments have different impacts on onset and progression of monocarpic senescence compared to wildtype *Arabidopsis* plants Columbia-0 (Col-0). The expression of the H_2_O_2_ sensor HyPER under the control of the 35S-CaMV promoter leads to constant and high expression of the HyPER protein and thereby scavenges H_2_O_2_. By directing HyPER to specific compartments, the H_2_O_2_ levels were specifically reduced there. While cytoplasmic and peroxisomal HyPER protein expression caused a delay in the onset of monocarpic senescence, chloroplastic HyPER expression retarded the progression of senescence

**Fig. 3:**
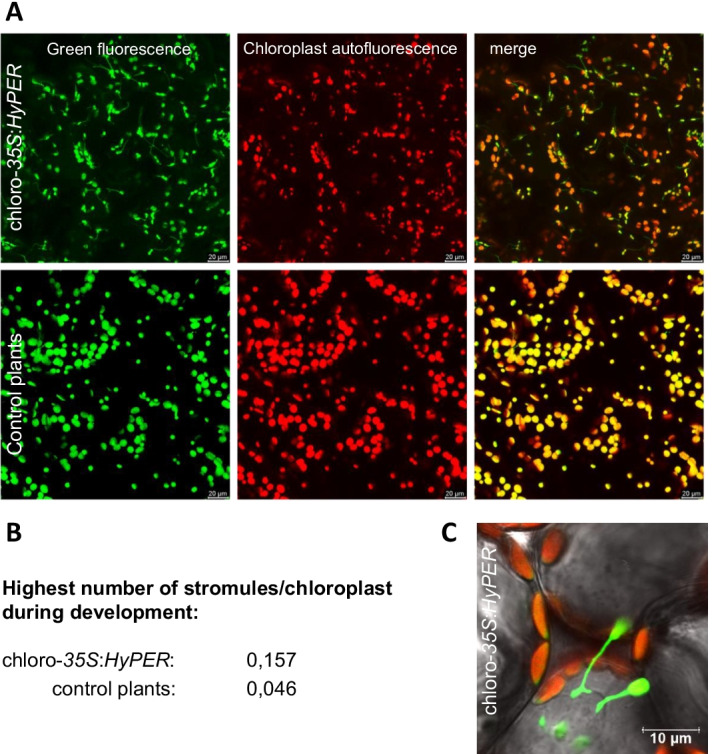
**A** An increase in the number of stromules formed by chloroplasts harboring the HyPER protein which scavenges H_2_O_2_ or a GFP which does not scavenge H_2_O_2_ (control plants) was observed. HyPER fluorescence (green) and autofluorescence of the chloroplasts (red) and a merge of these are presented. **B** The highest average number of stromules per chloroplasts in leaves of the chloro-35S:HyPER line and the control plants, which was counted over different developmental stages, is indicated. **C** Stromules are able to contact the nucleus shown in a merged microscopic image of transmitted light, HyPER fluorescence (green) and autofluorescence of the chloroplasts (red)

**Fig. 4:**
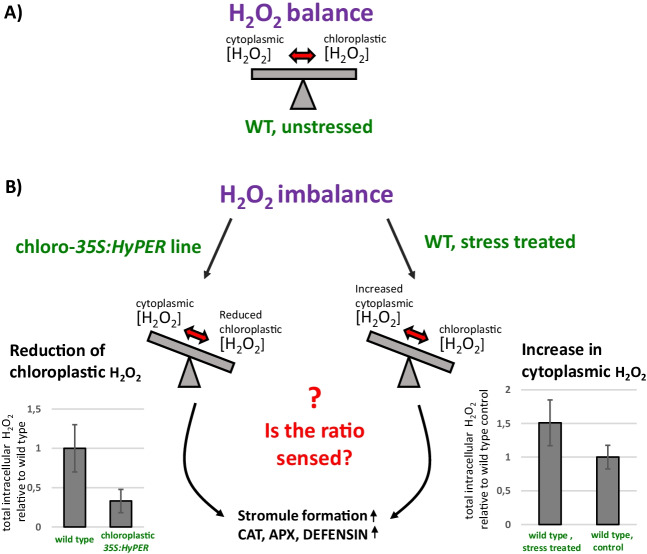
Model for H_2_O_2_ sensing. **A** Balanced H_2_O_2_ contents in wild type plants (WT) in normal situations without exogenous stress. **B** Imbalanced H_2_O_2_ contents between chloroplast and cytoplasm in two different situations: (1) wild type versus HyPER expression in chloroplasts (left) or (2) wild type under control conditions versus wild type under stress conditions (right). Total intracellular H_2_O_2_ contents were measured by H2DCFDA and expressed relative to wild type or wild type control conditions. In both cases, H_2_O_2_ content is imbalanced in the same way and high stromule numbers as well as expression of genes induced by oxidative stress are observed, indicating that the ratio between the contents of the cytoplasm and the chloroplasts might be the parameter which is sensed by the cells. This is still a hypothesis and has to be further analyzed, e.g. in crossings between plants expressing HyPER in the cytoplasm and in the chloroplasts

Costa and coworkers created transgenic lines which express this sensor protein in defined cellular compartments [104]. Yet, by directing HyPER to different compartments and thereby scavenging H_2_O_2_ specifically in these compartments, we could observe that the senescence process is differentially affected in these transgenic lines: While a reduction in peroxisomal and cytosolic H_2_O_2_ levels led to a delay in the onset of leaf senescence, low chloroplastic H_2_O_2_ retarded progression of leaf senescence (Fig. 2) ([49], unpublished observations). Moreover, genome wide gene expression analyses of these plants uncovered severe differences in gene expression, which were also dependent on the developmental stage. Here, plants with low chloroplastic H_2_O_2_ show more severe and often even opposite changes in gene expression compared to plants with lower cytoplasmic H_2_O_2_, which appear to be more similar in gene expression to wild type plants. Strikingly, higher expression of *CAT* and *APX* genes in plants with low chloroplastic H_2_O_2_ was observed. Moreover, expression of defensin genes is increased under low chloroplastic H_2_O_2_ but decreased under low cytoplasmic H_2_O_2_ (unpublished observation). In wild type plants, the expression of the pathogen-related defensin genes and genes encoding H_2_O_2_ scavenging proteins CAT and APX is triggered by oxidative stress but not by low H_2_O_2_. In the same chain of evidence, formation of stromules clearly increased under low chloroplastic H_2_O_2_ (Fig. 3; unpublished observation), which, again, should be opposite and rise after SA or H_2_O_2_ treatment [80]. These opposing expression patterns together with the unexpected increase in stromule formation prompted us to speculate that not the actual H_2_O_2_ concentration is sensed by the plants but more likely the ratio between the cytoplasmic and the chloroplastic H_2_O_2_. This would mean that low chloroplastic H_2_O_2_ resembles “cytoplasmic” oxidative stress (Fig. 4). If our hypothesis of sensing the H_2_O_2_ ratio is correct, crossings between the two HyPer transgenic lines should be similar to wild type plants as now H_2_O_2_ would be low in both compartments. Presumably, defensin and *CAT/APX* gene expression, stromule formation, and senescence onset and progression will then be more reminiscent of wild type plants. However, how this ratio is sensed by the plants still has to be elucidated.

## Future perspectives

Reactive oxygen species (ROS) are inevitable by-products of many reactions of aerobic organisms, are produced in all subcellular compartments and play pivotal roles in the communication between all plant organelles [35, 105–107]. However, how specificity is achieved in H_2_O_2_ or ROS signaling in general is still an open question. Direct connections between different compartments such as stromules or peroxules enable small signaling molecules as well proteins or metabolites to be directly exchanged without passing through the cytoplasm. ROS, especially H_2_O_2_, can also pass through these tunnels to avoid the scavenging system of the cytoplasm imposing directionality in signaling. However, how the cells differentiate between stress-induced ROS signals and developmental ROS signals, e.g. the senescence-inducing H_2_O_2_ increase, is still not well understood. Localization of ROS production, especially for short-lived ROS, as well as duration of the production, might be part of this differentiation. However, as no “master receptors” for ROS are known so far, and a plethora of possible targets for each ROS is available, the complex sensing process still has to be elucidated. Improvement of the sensor proteins [108] as well as of the microscopic technologies, e.g. single molecule or super resolution microscopy, will increase resolution and will help to understand the signaling processes in more detail. Single cell transcriptomics and metabolomics will also contribute to a more holistic view of the outcome of ROS signaling. In addition, artificial intelligence highly improved our understanding of protein structures [109] and could help in the future to predict structural changes under different redox conditions. So far, an enormous experimental effort has been necessary to unravel the structures of approximately 100,000 unique proteins, which still is only a tiny fraction of the billions of existing proteins. In contrast to the explosion in available genomic sequences and gene expression data produced by the technical advances in sequencing methods, a similar expansion in knowledge on available protein structures has so far been prevented by the intrinsic challenge of experimental structure determination. AlphaFold2 is a novel machine learning approach that incorporates physical and biological knowledge about protein structure which is now capable of predicting protein structures to near experimental accuracy in a majority of cases and greatly outperforms other methods [109]. Changes of protein structures by different redox environments await integration in the next step. Therefore, it can be expected that these technological advances will foster our understanding of intracellular redox signaling during development and stress and will help to determine how specificity in signaling is achieved.

## Data Availability

Not applicable.

## Abbreviations

ROS: Reactive oxygen species
F_V_/F_M_: Photochemical capacity
RCBs: Rubisco-containing bodies
ATI-PS bodies: ATG8-interacting-protein1-plastid-associated bodies
SAVs: Senescence-associated vacuoles
CCVs: Chloroplast vesiculation-containing vesicles
TF: Transcription factor
APX: Ascorbate peroxidase
CAT: Catalase
GBF1: G-Box binding factor 1
AOX: Alternative oxidase
HyPER: Hydrogen peroxide sensor protein
roGFP: Redox-sensitive green fluorescent protein
RCD1: RADICAL-INDUCED CELL DEATH1 protein
NBT: Nitroblue tetrazolium
DAB: Diaminobenzidine
H2DCFDA: 2′,7′-Dichlorofluorescin diacetate
OxyR-RD: OxyR transcription factor regulatory domain of *Escherichia coli*
